# *De Novo* Assembly and Characterization of Narrow-Ridged Finless Porpoise Renal Transcriptome and Identification of Candidate Genes Involved in Osmoregulation

**DOI:** 10.3390/ijms16012220

**Published:** 2015-01-20

**Authors:** Rui Ruan, Ai-Huan Guo, Yu-Jiang Hao, Jin-Song Zheng, Ding Wang

**Affiliations:** 1 The Key Laboratory of Aquatic Biodiversity and Conservation of Chinese Academy of Sciences, Institute of Hydrobiology of Chinese Academy of Sciences, Wuhan 430072, China; E-Mails: ruan.rui@163.com (R.R.); guoaihuan10@126.com (A.-H.G.); hao.yj@ihb.ac.cn (Y.-J.H.); 2 University of Chinese Academy of Sciences, Beijing 100049, China

**Keywords:** renal transcriptome, RNA-seq, the narrow-ridged finless porpoise, osmoregulation, AQP2

## Abstract

During the evolutionary transition from land to water, cetaceans have undergone numerous critical challenges, with osmoregulation being the major one. Two subspecies of the narrow-ridged finless porpoise (*Neophocaena asiaeorientalis*), the freshwater Yangtze finless porpoise (*N. a. asiaeorientalis*, NAA) and the marine East Asian finless porpoise (*N*.* a*.* sunameri*, NAS), provide excellent subjects to understand the genetic basis of osmoregulatory divergence between freshwater and marine mammals. The kidney plays an important and well-established role in osmoregulation in marine mammals and thus, herein, we utilized RNA-seq to characterize the renal transcriptome and preliminarily analyze the divergence between the NAA and the NAS. Approximately 48.98 million clean reads from NAS and 49.40 million clean reads from NAA were obtained by RNA-Seq. And 73,449 (NAS) and 68,073 (NAA) unigenes were assembled. Among these annotations, 22,231 (NAS) and 21,849 (NAA) unigenes were annotated against the NCBI nr protein database. The ion channel complex GO term and four pathways were detected as relevant to osmoregulation by GO and KEGG pathway classification of these annotated unigenes. Although the endangered status of the study species prevented analysis of biological replicates, we identified nine differentially expressed genes (DEGs) that may be vital in the osmoregulation of the narrow-ridged finless porpoise and worthwhile for future studies. Of these DEGs, the differential expression and distribution of the aquaporin-2 (AQP2) in the collecting duct were verified using immunohistochemical experiments. Together, this work is the first report of renal transcriptome sequencing in cetaceans, and it will provide a valuable resource for future molecular genetics studies on cetacean osmoregulation.

## 1. Introduction

Osmotic stress was one of the most critical challenges for cetaceans, which underwent an evolutionary transition from land to water approximately 50 million years ago [1,2]. The kidney is the principal organ for maintaining water and electrolyte homeostasis, and plays an important, well-established role in osmoregulation via the formation and excretion of urine in mammals [3,4]. However, a few anatomical modifications (for example: reniculi) in the kidney of cetaceans do not seem to afford them any greater benefit than terrestrial mammals [3,5]. As a result, physiological modifications in kidneys are crucial for the survival of cetaceans [6]. To date, studies on the osmoregulatory mechanisms in cetaceans have been focused on the renal structure [5], the concentrations of ions in serum and urine [6], and the experiments of drinking, fasting and feeding [3]. These studies provided significant contributions to understand the water and electrolyte metabolism and the renal function in cetaceans, but the genetic basis of osmoregulation in cetaceans for adapting to the distinct marine or freshwater environments remains poorly explored [7].

Two subspecies of narrow-ridged finless porpoises (*Neophocaena* *asiaeorientalis*) live in Chinese waters: one is a freshwater subspecies (the Yangtze finless porpoise, *N*. *a*.* asiaeorientalis*) inhabiting the middle and lower reaches of the Yangtze River and adjoining Poyang and Dongting lakes, the other is a marine subspecies (the East Asian finless porpoise,* N*.* a*. *sunameri*) inhabiting the coastal waters of the Yellow Sea, the Bohai Sea and the northern part of the East China Sea [8,9]. Based on a phylogeography study of mitochondrial markers, it was inferred that finless porpoises may have expanded from the Yellow Sea to the Yangtze River approximately 22,000 years ago [10], which is a very recent population expansion event. In addition, the osmolality of seawater is more than 1000 mosmol/kg, whereas that of freshwater is less than 25 mosmol/kg [11]; and also the urinary osmolality of the Yangtze finless porpoise significantly differs from that of the East Asian finless porpoise (934.6 and 1223.8 mosmol/kg, respectively) [6]. These data demonstrate that the two subspecies exhibit different physiological levels of urinary osmolality, indicative of distinct osmoregulatory mechanisms [6]. Thus the narrow-ridged finless porpoise would be an excellent subject to explore the genetic mechanism of osmoregulatory divergence between marine mammals living in distinct environments.

In this study, high-throughput RNA sequencing was performed to characterize the renal transcriptome of a freshwater Yangtze finless porpoise (NAA) and a marine East Asian finless porpoise (NAS). Four pathways related to osmoregulation or urine formation were found through KEGG pathway classification on annotated unigenes. In comparative differential expression analysis, we could not distinguish the exact causes of differential expression between the two samples (one sample for each population), but nine differentially expressed genes (DEGs) were found, which were probably involved in osmoregulation and deserved further study. One of these DEGs, aquaporin-2 (AQP2), was verified using immunohistochemical experiments. In addition, the description of the transcriptomes in this study represents a good resource for future studies.

## 2. Results and Discussion

### 2.1. RNA Sequencing, de Novo Assembly and Functional Annotation

A total of 48.98 and 49.40 million clean reads were obtained from the NAS and NAA samples, respectively, following filtering out poor-quality and adapter-related reads as well as reads with a >10% N content (where N represents bases that cannot be determined), and these reads have been deposited in the NCBI Sequence Read Archive (accession numbers: SRX387810 for NAA and SRX387808 for NAS). We obtained three assemblies, which included 114,057 transcripts for NAS, 105,813 transcripts for NAA and 165,547 transcripts for the combined assembly (Table 1). Consequently, we constructed three unigene sets, containing 73,449 unigenes for NAS, 68,073 unigenes for NAA and 103,077 unigenes for the combined assembly, with the average lengths of 740, 735 and 685 bp, respectively (Table 1). The assembled unigenes have been deposited in the NCBI Transcriptome Shotgun Assembly (TSA) database under the accession number of GBYL00000000 for NAA and GBYP00000000 for NAS. The versions described in this paper are the first version. The size distributions of transcripts and unigenes for NAS, NAA and combined assembly are shown in Figure S1.

**Table 1:** Summary of the *de novo* assembly of clean reads. NAA: the Yangtze finless porpoise; NAS: the East Asian finless porpoise.

Parameters	NAA	NAS	Combined
Number of assembled transcripts	105,813	114,057	165,547
Number of unigenes	68,073	73,449	103,077
Mean unigene length (bp)	735	740	685
Maximum unigene length (bp)	19,040	12,579	17,179
Minimum unigene length (bp)	201	201	201
N50 (unigenes) (bp)	1389	1447	1227
N90 (unigenes) (bp)	270	269	260

N50/N90: the length of unigenes for which the cumulative size is not shorter than 50% or 90% of the total size of all unigenes, when all unigenes are sorted from longest to shortest.

**Figure 1:**
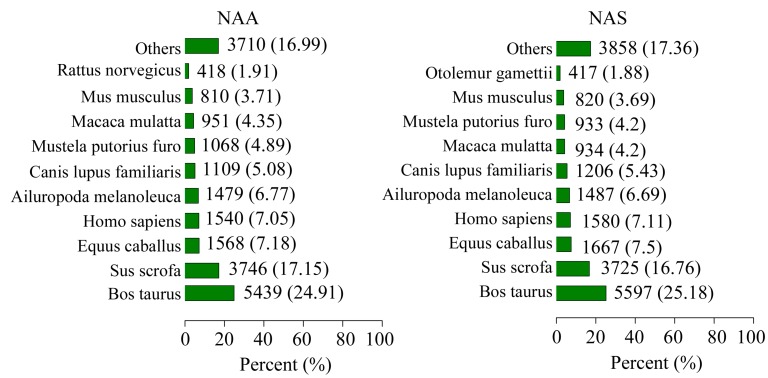
Distributions of the species similarity in NAA and NAS. NAA: the Yangtze finless porpoise; NAS: the East Asian finless porpoise.

A total of 22,231 unigenes from 73,449 NAS unigenes and 21,849 unigenes from 68,073 NAA unigenes, respectively, were successfully annotated by performing NCBI BLAST search against the NCBI nr protein database using an E-value < 10^−5^ (Table 2, Table S1). The distribution of the species similarity based on the successfully annotated unigenes revealed (Figure 1), that the highest percentages of NAS and NAA unigenes were matched to *Bos taurus* (25.18% and 24.91%), followed by *Sus scrofa* (16.76% and 17.15%). This result was in line with the research that cetaceans have originated from early Artiodactyls [12].

**Table 2:** Summary of unigenes annotated in important public databases. NAA: the Yangtze finless porpoise; NAS: the East Asian finless porpoise.

Public Database	Number of Annotated Unigene		Percentage (%) of Assembled Unigenes	
	NAA	NAS	NAA (68,073)	NAS (73,449)
NR	21,849	22,231	32.10	30.27
NT	35,260	36,661	51.80	49.91
SwissProt	20,485	20,639	30.10	28.10
GO	17,550	18,163	25.78	24.73
KOG	11,555	11,867	16.97	16.16
KEGG	11,165	11,163	16.40	15.20

Gene Ontology (GO) and Eukaryotic of Orthologous Groups (KOG) are often used to annotate and analyze gene function in any organism [13,14]. Unigenes, 17,550 for NAA and 18,163 for NAS, were assigned to GO terms (Table 2). In the cellular component category of GO, 43 unigenes for NAA and 54 unigenes for NAS were assigned in the ion channel complex term, respectively (Table S2). Ion channels play an important role in the process of ion transport, so the knowledge of ion channels would contribute to understanding osmoregulation in cetaceans. And 11,555 unigenes for NAA and 11,867 unigenes for NAS were clustered into 26 functional categories of KOG among which “Signal Transduction” represented the largest group in NAA and NAS (2393 unigenes for NAA and 2457 unigenes for NAS) (Table S3). This result did not accord with the case in the Indo-Pacific humpback dolphin (*Sousa chinensis*) leucocyte transcriptome that “general function prediction only” represented the largest group followed by “translation, ribosomal structure and biogenesis” [15]. The main reason might be that different tissues or organs play different roles in the organism, so the expressed genes also might be divergent.

**Figure 2:**
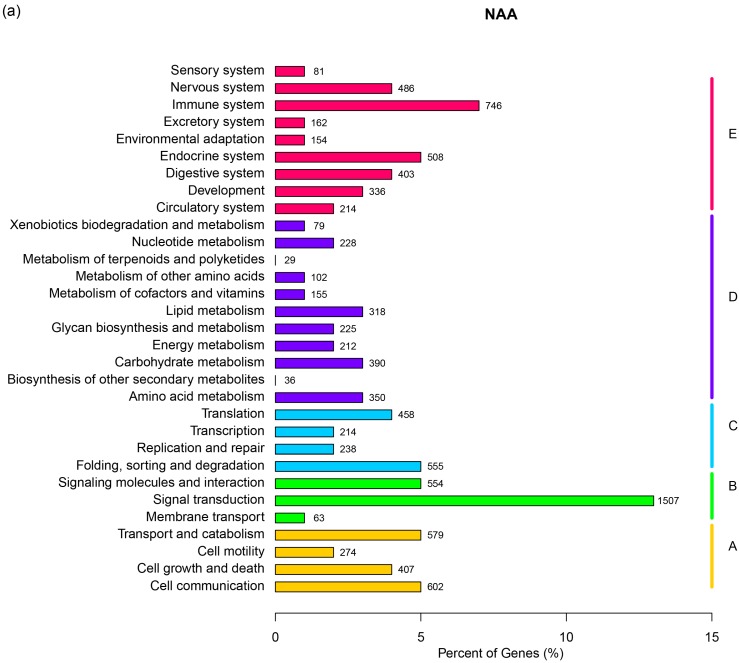

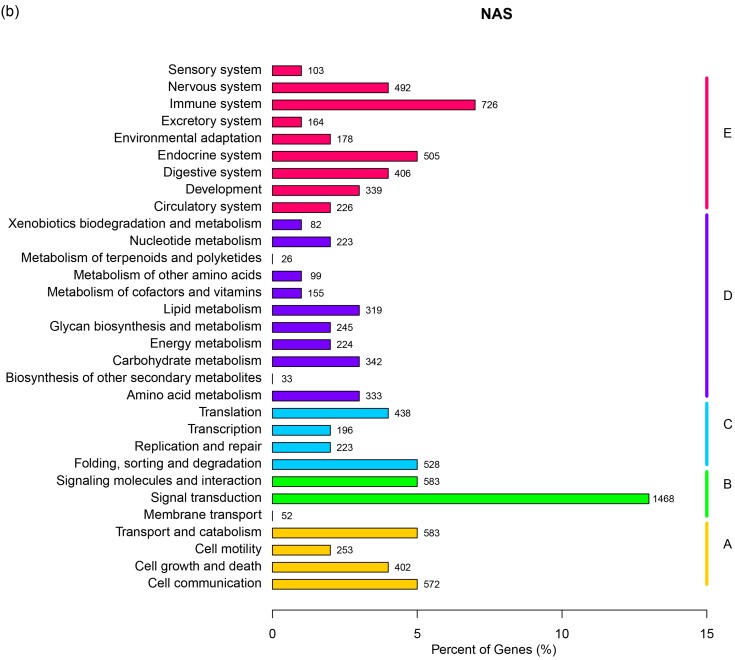
KEGG pathway classification of unigenes. (**a**) NAA; (**b**) NAS. The KEGG pathways were categorized into five groups, A: Cellular process, B: Environmental information processing, C: Genetic information processing, D: Metabolism and E: Organismal system. KEGG: Kyoto Encyclopedia of Genes and Genomes; NAS: the East Asian finless porpoise; NAA: the Yangtze finless porpoise.

To identify the biological pathways that are active in the kidney of the narrow-ridge finless porpoise, KEGG (Kyoto Encyclopedia of Genes and Genomes) pathways classification was performed. Unigenes for NAA (11,165) were grouped into 241 pathways, and unigenes for NAS (11,163) were also grouped into 241 pathways (Table 2, Table S4). On the basis of the unigenes involved in the KEGG pathways, they were classified into five categories which included cellular processes, environmental information processing, genetic information processing, metabolism and organismal systems. The distributions of KEGG pathways for NAA and NAS were extremely similar (Figure 2a,b). As shown in Figure 2, the signal transduction pathway had the largest number of unigenes in NAA and NAS among the pathways. Clearly, however, the osmoregulatory process was mainly involved with two pathways: the endocrine system and the excretory system [3]. In NAA, the numbers of unigenes in the endocrine and excretory systems were 508 and 162, respectively; in NAS, there were 505 unigenes for the endocrine system and 164 unigenes for the excretory system (Figure 2a,b). In the endocrine system, the renin-angiotensin system was found which regulates blood pressure and water balance [16]. We found that aldosterone-regulated sodium reabsorption, proximal tubule bicarbonate reclamation and vasopressin-regulated water reabsorption were in the excretory system. In Table 3, the numbers of unigenes were listed, which were assigned to the renin-angiotensin system, the aldosterone-regulated sodium reabsorption, the proximal tubule bicarbonate reclamation and the vasopressin-regulated water reabsorption.

**Table 3:** The number of unigenes involved in the osmoregulatory process by KEGG pathways classification analysis. KEGG: Kyoto Encyclopedia of Genes and Genomes; NAS: the East Asian finless porpoise; NAA: the Yangtze finless porpoise.

KEGG Pathway	The Number of Unigenes in NAA	The Number of Unigenes in NAS
Renin-angiotensin system	18	22
Aldosterone-regulated sodium reabsorption	41	35
Proximal tubule bicarbonate reclamation	18	19
Vasopressin-regulated water reabsorption	50	59

Previous studies showed that the variation in the renal morphology of cetaceans seems incapable to offer them any greater benefit for adapting to a hyperosmotic environment than terrestrial mammals [3]. This suggests that the conventional mechanisms of urine concentration (for example: hormonal regulation) might be vital for marine mammals [3]. At present, very limited information on the osmoregulation of cetaceans is known. The renin-angiotensin system, the aldosterone-regulated sodium reabsorption, the vasopressin-regulated water reabsorption and the proximal tubule bicarbonate reclamation found in this work might provide a significant resource to understand or to explore cetacean osmoregulation. Especially for hormonal regulation of urine concentration, in the future we could examine hormone levels in serum of the narrow-ridged finless porpoises under divergent osmotic waters, to provide further information to understand the mechanism of maintaining water balance and electrolyte homeostasis.

### 2.2. Preliminary Comparative Analysis of Transcriptomes between NAA and NAS

Following reciprocal BLASTn searches and screening of putative orthologous genes against the Swiss-Prot dataset via BLASTx, 5095 pairs of orthologous genes were retained for additional analyses. Based on the predicted coding regions, 4840 pairs of coding sequences (CDSs) greater than 150 bp in length were retained for subsequent analysis.

To determine genes under positive selection between the NAA and NAS orthologs, the Ka/Ks ratios were estimated based on the alignment of 4840 pairs of predicted orthologous CDSs. Of the 4840 pairs, only eight pairs of orthologous sequences showed Ka/Ks values larger than one, but their *p* values were larger than 0.05 (Table S5). It was not surprise that there was no significant divergence observed at the coding sequence level about the response to osmotic stress between NAA and NAS, due to the divergence about 22,000 years ago between the two subspecies. This result suggested that the changes in gene function for the narrow-ridged finless porpoise were insufficient to explain the adaptation to hypoosmotic environment. Perhaps, differential gene expression may play a crucial role in population survival during the colonization of new environments [17].

**Figure 3:**
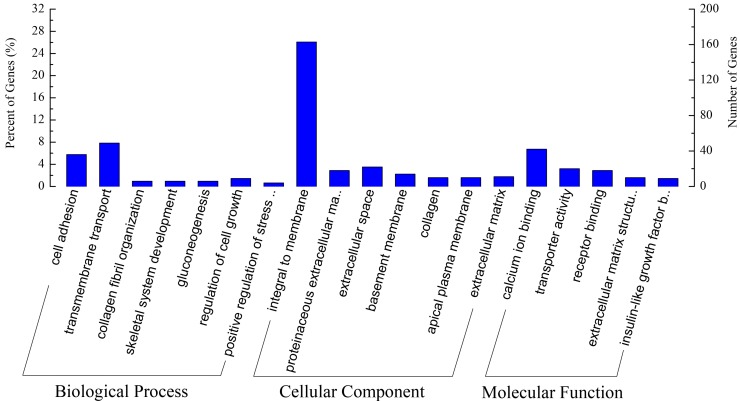
GO enrichment analysis of DEGs between NAS and NAA. GO: Gene Ontology; DEGs: differentially expressed genes; NAS: the East Asian finless porpoise; NAA: the Yangtze finless porpoise.

A total of 785 differentially expressed sequences were obtained using the DEGseq software, but only 656 DEGs (including 388 up-regulated DEGs and 268 down-regulated DEGs in NAS) were successfully annotated (Table S6). And the GO enrichment of these annotated DEGs was analyzed using GOseq software (Table S7). We found that the GO term integral to membrane showed the most significant enrichment and also presented the greatest number of DEGs among the enriched GO terms (Figure 3), with 163 DEGs being significantly enriched in this term.

We used the KOBAS software to further explore the distribution of the DEGs in the KEGG pathways (Table S8). We found one significant enrichment pathway involved in the process of urine formation, which was the proximal tubule bicarbonate reclamation. However, the renin-angiotensin system (RAS) was not significantly enriched (Q-value = 0.063), which also indicated some DEGs in the process of urine formation. Intriguingly, integral to membrane and urine formation-related pathways are probably associated with the osmotic challenge, because the biomembrane would be firstly subjected to the osmotic stress in hyperosmotic or hypoosmotic environments, and urine formation plays a vital role in the process of maintaining water and electrolyte homeostasis [3]. In particular, some of the genes involved in the RAS exhibited cetacean-specific amino acid changes in comparison with terrestrial mammals [18]. We also found that two DEGs related to urine formation were enriched in the GO term integral to membrane: urea transporter 2 (UT2) and aquaporin-2 (AQP2). UT2 and AQP2 were subjected to positive selection in the major cetacean clades and might contribute to the concentration of urine and the maintenance of water balance [7]. We also identified nine DEGs potentially involved in urine formation and regulation of water and electrolyte balance in the kidney from the most enriched GO term (integral to membrane) and the enriched KEGG pathway of RAS (Table 4). The functions of the nine genes were listed in Table 4.

**Table 4:** DEGs related to urine formation were selected from the GO term integral to membrane and the KEGG pathway of the renin-angiotensin system. DEGs: differentially expressed genes; GO: Gene Ontology; KEGG: Kyoto Encyclopedia of Genes and Genomes.

Gene-ID	Gene Name	log2(Fold-Change)	Function in the Kidney	References
**comp46908_c0**	angiotensin I converting enzyme precursor	−3.71	Angiotensin I (ANG I) converting enzyme can convert angiotensin I to angiotensin II via the removal of two amino acids.	[19]
**comp46499_c0**	type-1 angiotensin II receptor (ANGR II)	3.32	Angiotensin II (ANG II) has a greater effect on efferent arterioles than on afferent arterioles by binding to the type-1 angiotensin II receptor. Though it decreases blood flow, it raises systemic arterial blood pressure while increasing glomerular pressure and the filtration fraction and maintaining the glomerular filtration rate. Ultimately, decreased hydrostatic pressure and increased oncotic pressure in the peritubular capillaries will facilitate the reabsorption of tubular fluid.	[20,21,22]
**comp45455_c0**	angiotensinogen	−2.52	Angiotensinogen is the ultimate precursor of angiotensin II and is cleaved by renin to generate angiotensin I.	[19]
**comp44526_c0**	sodium/hydrogen exchanger 3 (NHE3) precursor	−2.61	Sodium/hydrogen exchanger 3 is responsible for Na^+^ reabsorption and H^+^ secretion and is distributed throughout the apical membrane of the proximal tubule and the thick ascending limb.	[23]
**comp48685_c0**	aquaporin-2 (AQP2)	2.30	Aquaporin-2 is a water channel that localizes to the apical membrane and intracellular vesicles of the collecting duct.	[24]
**comp16873_c0**	aquaporin-3 (AQP3)	2.10	Aquaporin-3 is a water channel that localizes to the basolateral membrane of the collecting duct.	[24]
**comp46970_c0**	urea transporter 2 (UT2)	2.42	Urea transporter 2 mediates the diffusion of urea across the wall of the inner medullary collecting duct and thin descending limb of the loop of Henle and plays an important role in the maintenance of high osmotic pressure in the inner medulla.	[25,26]
**comp47244_c0**	solute carrier family 12 member 1	1.02	Solute carrier family 12 member 1 encodes a Na^+^–K^+^–Cl^−^ cotransporter distributed throughout the luminal membrane of the thick ascending limb of the loop of Henle.	[27,28]
**comp46740_c1**	atrial natriuretic peptide receptor 1 (ANPR1) precursor	1.59	Atrial natriuretic peptide binds the atrial natriuretic peptide receptor 1 and only inhibits the reabsorption of sodium in the inner medullary collecting duct and suppresses the secretion of renin, aldosterone and vasopressin but also directly affects the glomerular filtration rate.	[29,30]

Since our experimental subjects are strictly protected by laws in China, and the Yangtze finless porpoise is a critically endangered population in the IUCN Red Data Book [31], we could only obtain the fresh kidney samples right after the accidental deaths of two animals. We were unable to conduct biological replications in each subspecies, which prevented us from drawing strong conclusions of osmoregulatory mechanisms. To minimize this effect, we strictly normalized our transcriptome data, and conducted analysis by a more conservative program. More importantly, our results of differential expression were confirmed by enrichment analysis and the following AQP2 immunohistochemical evidence. However, we still could not distinguish the exact reason for the result of differential expression (e.g., different ages, different sexes, or different living environments) due to the sampling limitation. The comparative differential expression between NAA and NAS in this work was merely a preliminary study on osmoregulation. In spite of a lack of biological replications, the comparative analysis between the two transcriptomes presented a good resource for further studies. The results indicated that these genes should be vital to osmoregulation in the narrow-ridged finless porpoise and could provide important information for further studies of osmoregulation. Four of nine DEGs were related to hormonal regulation of urine concentration. Especially the DEG, atrial natriuretic peptide receptor 1 (ANPR1) precursor, indicated that atrial natriuretic peptide (ANP) would play a role in osmoregulation of cetaceans. Till now, ANP has not yet been examined, so it should be taken into account in future studies.

### 2.3. AQP2 Protein Expression

**Figure 4:**
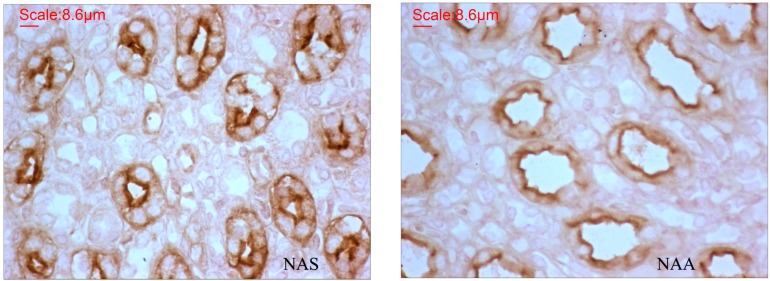
Distributions of AQP2 protein in the medulla of the renicule in NAS and NAA. The dark yellow color shows that AQP2 protein localized to the collecting ducts. Magnification: 40×. AQP2: aquaporin-2; NAS: the East Asian finless porpoise; NAA: the Yangtze finless porpoise.

To further explore whether the differential expression of urine formation-related genes has an effect on protein levels, and also to verify the result of differential expression analysis, we performed immunohistochemical experiments to detect the AQP2 protein in the NAS and NAA. We found that AQP2 protein was indeed distributed in the collecting duct (Figure 4) and that this protein showed significant differences between NAS and NAA (*p* < 0.05). The measurement parameters were shown in Table 5. The higher level of AQP2 protein found in the East Asian finless porpoise was consistent with the gene expression results.

**Table 5:** The IOD, density mean and area sum values of AQP2 protein in NAS and NAA. IOD: integrated optical density, NAS: the East Asian finless porpoise, NAA: the Yangtze finless porpoise.

	IOD Mean ± SE	Density Mean ± SE	Area Sum Mean ± SE
NAS	84,122.2 ± 5388.5	26.4 ± 1.3	250,258.3 ± 15,482.7
NAA	36,534.0 ± 2752.8	17.0 ± 1.2	112,554.0 ± 8457.2
Fold-change	2.30	1.55	2.22
*p*	<0.0001	<0.0001	<0.0001

It has been verified that AQP2 protein is distributed on the apical membrane of the collecting ducts of the cetaceans [32], and AQP2 in terrestrial mammals is responsible for the reabsorption of water in collecting ducts [33]. Thus, the higher level of AQP2 expression would be conducive to reabsorb water in the marine finless porpoise. It is known in terrestrial mammals that AQP2 protein in collecting ducts is activated by arginine-vasopressin (AVP) [34]. But, reports about AVP in cetaceans were still rare [32]. In this work, the vasopressin-regulated water reabsorption pathway was found. It suggested that AVP would regulate AQP2 in the process of water reabsorption in cetaceans. However, due to the sampling limitation of this work, further studies are necessary to verify this result.

## 3. Materials and Methods

### 3.1. Sample Preparation and RNA Sequencing

On the IUCN Red List of Threatened Species, the Yangtze finless porpoise (NAA) is listed as a critically endangered population [31], while the East Asian finless porpoise (NAS) is listed as a vulnerable population [35]. Since they are under protection and are forbidden to kill by laws in China, animals could be sampled only after they were dead by accident.

On 16 February 2012, a Yangtze finless porpoise (NAA) was found entangled in a gill net in the Honghu section of the main channel of the Yangtze River. This animal was a male calf, 95 cm in length. On 10 May 2012, an East Asian finless porpoise (NAS) was accidentally killed by a fishing net in the Bohai Sea. This porpoise was a female calf with a body length of 76 cm. Necropsies were conducted on both porpoises within two hours, and kidney samples were collected and preserved in liquid nitrogen. All sampling was conducted in accordance with the Regulations of the People’s Republic of China for the Implementation of Wild Aquatic Animal Protection (published in 1993) and adhered to all Chinese ethical guidelines and legal requirements.

Total RNA was extracted from each sample using the RNAprep Pure Kit (TIANGEN, Beijing, China) according to the manufacturer’s protocol. We collected 60 μL (200 ng/μL) and 55 μL (359 ng/μL) total RNA from NAS and NAA, respectively. The RNA samples were assessed to ensure that they met the quantitative and qualitative requirements of RNA-seq [36,37,38] (Table S9). The two RNA samples were then sent to Beijing Novogene Bioinformation Technology Co., Ltd. (Beijing, China) for RNA sequencing. Parallel sequencing libraries were constructed using the IlluminaTruSeq™ RNA Sample Preparation Kit (Illumina, San Diego, CA, USA). The mRNA was purified from total RNA using poly-T oligo-attached magnetic beads. Fragmentation was carried out using divalent cations under elevated temperature in Illumina proprietary fragmentation buffer. First strand cDNA was synthesized using random oligonucleotides and SuperScript II. Second strand cDNA synthesis was subsequently performed using DNA Polymerase I and RNase H. Remaining overhangs were converted into blunt ends via exonuclease/polymerase activities and enzymes were removed. After adenylation of 3' ends of DNA fragments, Illumina PE adapter oligonucleotides were ligated to prepare for hybridization. In order to select cDNA fragments of preferentially 200 bp in length, the library fragments were purified with AMPure XP system (Beckman Coulter, Beverly, MA, USA). DNA fragments with ligated adaptor molecules on both ends were selectively enriched using Illumina PCR Primer Cocktail in a 10 cycle PCR reaction. Products were purified (AMPure XP system) and quantified using the Agilent high sensitivity DNA assay on the Agilent Bioanalyzer 2100 system (Agilent Technologies, Palo Alto, CA, USA). And then massively sequenced via parallelization on the Illumina Hiseq 2000 platform; 100 bp paired-end reads were generated.

### 3.2. Basic Processing of RNA-Seq Data and Gene Functional Annotation

After the sequenced reads had been translated from image data by Consensus Assessment of sequence and Variation (CASAVA) software of Illumina, the raw reads were first processed by NGSQC Toolkit (v2.2.2; http://www.nipgr.res.in/ngsqctoolkit.html). In this step, clean reads were obtained by removing reads containing adapter sequences, poly-N reads and low-quality reads (which contain above 50% bases with sequence quality less than 5) from the raw data. All downstream analyses were based on clean, high-quality data. Transcriptome assembly was accomplished using Trinity (v2012-10-05) [39], with min_kmer_cov set to 2 and all other parameters set to default values, including one combined assembly from the two sequenced datasets and two separate assemblies from the sequenced datasets from the two different samples. The first transcripts of each gene were pooled as “unigenes”, forming three pools in all.

The NAS and NAA unigenes were annotated against the Swiss-Prot protein database, NCBI NR protein and NT nucleotide databases, KOG and KEGG database using NCBI BLAST (2.2.27+) program (E-value < 10^−5^). Functional annotation of Gene Ontology (GO) was performed with Blast2GO (version 2.5) [40]. The unigene sequences were also aligned to the KOG (Eukaryotic Orthologous Groups of proteins) database to predict and classify possible functions. Pathway classifications were performed according to KEGG pathway database [41].

### 3.3. Comparative Analysis of Transcripts between NAA and NAS

We employed the reciprocal best hit method in BLASTn to identify potentially orthologous sequences between NAS and NAA. Pairs of unigenes that were each other’s best hit (E-value < 10^−5^) and those longer than 200 bp were retained. The reciprocal best hit method has been widely employed in comparative transcriptome studies to identify orthologous genes in closely related species [42,43,44]. This strategy has been shown to outperform more complex methods for identifying orthologous genes [45].

Since it is difficult to distinguish members of the same gene family in *de novo* transcriptome assemblies, paralogous genes could not be completely removed using the reciprocal best hit method [44]. To obtain pairs of orthologous genes with higher confidence, the aforementioned putative pairs of orthologous unigenes were further screened against the Swiss-Prot protein dataset. If a pair of putative orthologs mapped to different genes via BLASTx, they were removed; however, if they matched the same gene with an E-value < 10^−5^, an alignment length >200 bp and a similarity >90%, they were selected as orthologous genes without frameshifts and indels. Furthermore, Coding sequences (CDSs) with lengths of greater than 150 bp were retained. The coding sequences were extracted based on the result of alignment in which unigenes had been aligned in the SwissProt database by Blastx. If a unigene was not aligned on the SwissProt database, its coding sequence would be predicted by software Estscan (version 2.1) [46].

To identify genes undergoing selection at the transcriptome level, we estimated the rates of nonsynonymous (Ka) and synonymous (Ks) substitution between NAS and NAA. The Ka/Ks ratio indicates the mode of selection under which the coding sequence has been evolving: Ka/Ks > 1 is interpreted as signifying positive selection, while Ka/Ks < 1 indicates purifying selection [47]. KaKs_Calculator (version 1.2) was employed to estimate Ka/Ks using the YN method [48,49]. When genes with Ka/Ks > 1 were found, their sequences were checked manually and performed again by KaKs_Calculator software for guaranteeing their accuracy.

Since no reference genome was available, we identified differentially expressed genes (DEGs) between NAS and NAA using the unigenes from the combined assembly as the reference set. Clean data from each sample were mapped back onto the reference set. The read count for each gene was estimated based on the mapped results using RSEM [50]. The RPKM (Reads Per Kilobase per Million mapped reads) value for each unigene was then calculated based on the read count. The RPKM value, which takes into account the depth of the sequence and the length of the unigene, is currently the most commonly used method for estimating relative transcript levels [51].

To analyze differential gene expression between NAS and NAA, the NAS sample was used as the treatment group and the NAA sample as the control group. The NAA in the freshwater, as the terrestrial mammals, would retain salt and discharge water. DEGs were identified using DEseq [52] in the R package [53]. Under the assumption that a minority of genes with significantly differential expression levels in two samples from different conditions, DESeq can work without any replicates and produce more conservative results than edgeR [52]. To avoid sequencing artifacts, which could be generated by sequencing more highly expressed genes over lowly expressed ones, we used the trimmed mean of M (TMM) values to normalize the data from both samples [52,54,55]. We next detected differentially expressed genes between NAS and NAA samples using the MA plot-based method with random sampling model [53] with a Q-value < 0.005 and an absolute log_2_(fold-change) value > 1. The Q value is a measure of the “false discovery rate” (FDR) [56]. The DEGs were also used for GO and KEGG pathway enrichment analyses. GO enrichment analysis was performed using the Goseq based Wallenius non-central hyper-geometric distribution, which can adjust for gene-length bias in DEGs. KEGG pathway enrichment analysis was performed using KOBAS [57]. Significance was corrected using the Benjamini & Hochberg (BH) method [58].

### 3.4. AQP2 Protein Expression in the Kidney

Kidneys from the same two porpoises were cut into small blocks containing several renicules, and preserved in 10% neutral buffered formalin. The blocks were dehydrated by ethanol and xylene, then embedded in paraffin wax and longitudinally sliced (cortex-calyx direction) into 4 μm thick sections. The anti-rabbit AQP2 was bound to the peptide (RQSVELHSPQSLPRGSKA) in the intracellular (which corresponded to residues 254–271) rat AQP2 *C*-terminus (Accession number: P34080 in Uniprot). The procedure of the immunohistochemical experiment followed a previous study in the bottlenose dolphin and the Baird’s beaked whale [32].

All the sections were stained by the same researcher using exactly the same protocol of immunohistochemistry (IHC) in the same lab. Tissue sections were deparaffinized in xylene and rehydrated with gradual ethanol to water. The sections were boiled in 0.01 M citrate buffer (pH 6.0) in an autoclave at 121 °C for 10 min for antigen epitome’s retrieval. Endogenous peroxidase activity was blocked in a 3% H_2_O_2_ solution by incubation for 20 min at room temperature. After rinsing in PBS buffer and blocking in 10% non-immune goat serum for 30 min at room temperature, the sections were incubated with a 1:1000 dilution of affinity purified anti-rabbit antibody against a synthetic peptide corresponding to 15 carboxyl-terminal amino acid residues of rat AQP2 (Alomone Laboratories, Jerusalem, Israel) or non-immune rabbit serum (PBS) overnight at 4 °C. After washing with PBS, the sections were incubated with biotinylated second antibody to goat for rabbit IgG for 30 min at room temperature, and rinsed in PBS. Following incubation with biotin–avidin horseradish peroxydase complex for 20 min at room temperature and washed with PBS, the sections combined with antibodies were reacted with 0.02% diaminobezidine tetrahydrochloride (DAB, Boshide, Wuhan, China) for coloration in Mayer’s hematoxilin for 2 min, following by dehydration and coverslipping.

These sections were checked using optical microscope with a Plan 40×/0.70 objective (ZEISS, Jena, Germany). Digital images were captured by a CCD color video camera (TUCSEN, Fujian, China) with connection to a computer. The three-color channels of the camera were balanced by adjusting the microscope light intensity and camera gain and offset. Analysis was carried out with an observer blinded to the experimental protocol. Five sections per individual and ten fields per section of the same magnification were utilized for quantitative analysis. The mean optical densities of protein were measured using the Image-Pro-Plus 6.0 analysis system in each section [59,60].

The measurement parameters included density mean, area sum and IOD. The optical density was calibrated and the area of interest was set as: hue, 0–30; saturation, 0–255; intensity, 0–255. The time of analysis was greatly reduced by using a macro. T-test was performed on independent samples to compare the differences of AQP2 protein-level between marine and freshwater finless porpoise with SPSS 13.0.

## 4. Conclusions

Osmoregulation in cetaceans has been investigated for over a century, however the underlying mechanisms of maintaining water and electrolyte homeostasis remains unclear [3]. The kidney is an important organ in the process of regulating the excretion and resorption of water and solutes, thus characterization and comparative analysis of renal transcriptomes between cetaceans under distinct osmotic stresses would probably provide important insights into the genetic basis of osmoregulation. In this study, we characterized the renal transcriptomes of two narrow-ridge finless porpoises (one from freshwater subspecies, and the other from marine subspecies), and identified a few specific gene families involved in osmoregulation including the renin-angiotensin system, the aldosterone-regulated sodium reabsorption, the proximal tubule bicarbonate reclamation and the vasopressin-regulated water reabsorption. Although, in the differential expression analysis, we could not exclude noises from different ages, sexes, or health status* etc.*, the nine DEGs have potential in future osmoregulatory studies in cetaceans, which might also play vital roles in adaptation to distinct freshwater and marine environments.

This work is the first report of renal transcriptome sequencing in cetaceans, and will provide a valuable resource for future molecular genetics studies in cetacean osmoregulation. Many further systematic studies related to osmoregulatory mechanisms in cetaceans are still necessary and particularly, studies incorporating hormonal and ion channel component would be of interest. Also a comparison of osmoregulatory patterns between freshwater and sea cetaceans would provide instructive information about the maintenance of water balance and electrolyte homeostasis in marine mammals. In addition, because blood samples are more easily obtained through a non-invasive way than any other tissues, and since the blood transcriptome could also provide important information to understand the osmoregulatory mechanisms of hormones and ion channels in the blood, comparative studies of blood transcriptomes between the East Asian finless porpoise (NAS) and the Yangtze finless porpoise (NAA) should be carried out in the near future.

## Supplementary Materials

Supplementary materials can be found at: http://www.mdpi.com/1422-0067/16/01/2220/s1.

## Appendix

The clean reads for the East Asian finless porpoise (NAS) and the Yangtze finless porpoise (NAA) are now available in the NCBI Sequence Read Archive under the accession numbers: SRX387810 for NAA and SRX387808 for NAS. The assembled sequences have been deposited in the NCBI Transcriptome Shotgun Assembly (TSA) database under the accession number of GBYL00000000 for NAA and the accession number of GBYP00000000 for NAS. The versions described in this paper are the first version.

## References

[1] Gatesy J., O’Leary M.A. (2001). Deciphering whale origins with molecules and fossils. Trends Ecol. Evol..

[2] Thewissen J.G.M., Cooper L.N., Clementz M.T., Bajpai S., Tiwari B.N. (2007). Whales originated from aquatic artiodactyls in the Eocene epoch of India. Nature.

[3] Ortiz R.M. (2001). Osmoregulation in marine mammals. J. Exp. Biol..

[4] Grunewald R.W., Kinne R.K. (1999). Osmoregulation in the mammalian kidney: The role of organic osmolytes. J. Exp. Zool..

[5] Pfeiffer C. (1997). Renal cellular and tissue specializations in the bottlenose dolphin (*Tursiops truncatus*) and beluga whale (*Delphinapterus leucas*). Aquat. Mamm..

[6] Guo A., Hao Y., Wang J., Zhao Q., Wang D. (2014). Concentrations of osmotically related constituents in plasma and urine of finless porpoise (*Neophocaena asiaeorientalis*): Implications for osmoregulatory strategies for marine mammals living in freshwater. Zool. Stud..

[7] Xu S., Yang Y., Zhou X., Xu J., Zhou K., Yang G. (2013). Adaptive evolution of the osmoregulation-related genes in cetaceans during secondary aquatic adaptation. BMC Evol. Biol..

[8] Wang J., Frasier T., Yang S., White B. (2008). Detecting recent speciation events: The case of the finless porpoise (genus *Neophocaena*). Heredity.

[9] Wang J.Y., Yang S.C., Wang B.J., Wang L.S. (2010). Distinguishing between two species of finless porpoises (*Neophocaena phocaenoides* and *N. asiaeorientalis*) in areas of sympatry. Mammalia.

[10] Yang G., Guo L., Bruford M.W., Wei F.W., Zhou K.Y. (2008). Mitochondrial phylogeography and population history of finless porpoises in Sino-Japanese waters. Biol. J. Linn. Soc..

[11] Janech M.G., Chen R., Klein J., Nowak M.W., McFee W., Paul R.V., Fitzgibbon W.R., Ploth D.W. (2002). Molecular and functional characterization of a urea transporter from the kidney of a short-finned pilot whale. Am. J. Physiol.-Regul. Integr. Comp. Physiol..

[12] Gingerich P.D., Ul Haq M., Zalmout I.S., Khan I.H., Malkani M.S. (2001). Origin of whales from early artiodactyls: Hands and feet of Eocene Protocetidae from Pakistan. Science.

[13] Consortium G.O. (2004). The Gene Ontology (GO) database and informatics resource. Nucleic Acids Res..

[14] Tatusov R.L., Natale D.A., Garkavtsev I.V., Tatusova T.A., Shankavaram U.T., Rao B.S., Kiryutin B., Galperin M.Y., Fedorova N.D., Koonin E.V. (2001). The COG database: New developments in phylogenetic classification of proteins from complete genomes. Nucleic Acids Res..

[15] Gui D., Jia K., Xia J., Yang L., Chen J., Wu Y., Yi M. (2013). *De novo* assembly of the indo-pacific humpback dolphin leucocyte transcriptome to identify putative genes involved in the aquatic adaptation and immune response. PLoS One.

[16] Fyhrquist F., Saijonmaa O. (2008). Renin-angiotensin system revisited. J. Intern. Med..

[17] Pavey S.A., Collin H., Nosil P., Rogers S.M. (2010). The role of gene expression in ecological speciation. Ann. N. Y. Acad. Sci..

[18] Yim H.S., Cho Y.S., Guang X., Kang S.G., Jeong J.-Y., Cha S.-S., Oh H.-M., Lee J.-H., Yang E.C., Kwon K.K. (2014). Minke whale genome and aquatic adaptation in cetaceans. Nat. Genet..

[19] Griendling K.K., Murphy T., Alexander R.W. (1993). Molecular biology of the renin-angiotensin system. Circulation.

[20] Navar L., Rosivall L. (1984). Contribution of the renin-angiotensin system to the control of intrarenal hemodynamics. Kidney Int..

[21] Navar L.G., Harrison-Bernard L.M., Nishiyama A., Kobori H. (2002). Regulation of intrarenal angiotensin II in hypertension. Hypertension.

[22] Paul M., Mehr A.P., Kreutz R. (2006). Physiology of local renin-angiotensin systems. Physiol. Rev..

[23] Bobulescu I.A., Moe O.W. (2009). Luminal Na^+^/H^+^ exchange in the proximal tubule. Pflug. Arch.-Eur. J. Physiol..

[24] Nielsen S., Agre P. (1995). The aquaporin family of water channels in kidney. Kidney Int..

[25] Sands J.M. (1999). Regulation of renal urea transporters. J. Am. Soc. Nephrol..

[26] Bagnasco S.M. (2005). Role and regulation of urea transporters. Pflug. Arch.-Eur. J. Physiol..

[27] Quaggin S., Payne J., Forbush B., Igarashi P. (1995). Localization of the renal Na–K–Cl cotransporter gene (Slc12a1) on mouse Chromosome 2. Mamm. Genome.

[28] Delpire E., Kaplan M., Plotkin M., Hebert S. (1996). The Na–(K)–Cl cotransporter family in the mammalian kidney: Molecular identification and function(s). Nephrol. Dial. Transplant..

[29] Ballermann B., Brenner B. (1987). Atrial natriuretic peptide and the kidney. Am. J. Kidney Dis..

[30] Zeidel M.L. (1990). Renal actions of atrial natriuretic peptide: Regulation of collecting duct sodium and water transport. Annu. Rev. Physiol..

[31] Wang D., Turvey S.T., Zhao X., Mei Z. *Neophocaena Asiaeorientalis* ssp. *Asiaeorientalis*. IUCN 2013. IUCN Red List of Threatened Species.

[32] Suzuki M., Endo N., Nakano Y., Kato H., Kishiro T., Asahina K. (2008). Localization of aquaporin-2, renal morphology and urine composition in the bottlenose dolphin and the Baird’s beaked whale. J. Comp. Physiol. B.

[33] Frøkiaer J., Li C., Shi Y., Jensen A., Praetorius H., Hansen H., Topcu O., Sardeli C., Wang W., Kwon T.-H. (2002). Renal aquaporins and sodium transporters with special focus on urinary tract obstruction. APMIS Suppl..

[34] Noda Y., Sasaki S. (2006). Regulation of aquaporin-2 trafficking and its binding protein complex. Biochim. Biophys. Acta Biomembr..

[35] Wang J.Y., Reeves R. *Neophocaena* *Asiaeorientalis*. IUCN 2013. IUCN Red List of Threatened Species.

[36] Fleige S., Pfaffl M.W. (2006). RNA integrity and the effect on the real-time qRT-PCR performance. Mol. Asp. Med..

[37] Garbett K., Ebert P.J., Mitchell A., Lintas C., Manzi B., Mirnics K., Persico A.M. (2008). Immune transcriptome alterations in the temporal cortex of subjects with autism. Neurobiol. Dis..

[38] Schroeder A., Mueller O., Stocker S., Salowsky R., Leiber M., Gassmann M., Lightfoot S., Menzel W., Granzow M., Ragg T. (2006). The RIN: An RNA integrity number for assigning integrity values to RNA measurements. BMC Mol. Biol..

[39] Grabherr M.G., Haas B.J., Yassour M., Levin J.Z., Thompson D.A., Amit I., Adiconis X., Fan L., Raychowdhury R., Zeng Q. (2011). Full-length transcriptome assembly from RNA-Seq data without a reference genome. Nat. Biotechnol..

[40] Götz S., García-Gómez J.M., Terol J., Williams T.D., Nagaraj S.H., Nueda M.J., Robles M., Talón M., Dopazo J., Conesa A. (2008). High-throughput functional annotation and data mining with the Blast2GO suite. Nucleic Acids Res..

[41] Kanehisa M., Goto S. (2000). KEGG: Kyoto encyclopedia of genes and genomes. Nucleic Acids Res..

[42] Barreto F.S., Moy G.W., Burton R.S. (2011). Interpopulation patterns of divergence and selection across the transcriptome of the copepod *Tigriopus californicus*. Mol. Ecol..

[43] Elmer K.R., Fan S., Gunter H., Jones J., Boekhoff S., Kuraku S., Meyer A. (2010). Rapid evolution and selection inferred from the transcriptomes of sympatric crater lake cichlid fishes. Mol. Ecol..

[44] Wang X.W., Luan J.B., Li J.M., Su Y.L., Xia J., Liu S.S. (2011). Transcriptome analysis and comparison reveal divergence between two invasive whitefly cryptic species. BMC Genomics.

[45] Altenhoff A.M., Dessimoz C. (2009). Phylogenetic and functional assessment of orthologs inference projects and methods. PLoS Comput. Biol..

[46] Iseli C., Jongeneel C.V., Bucher P. (1999). ESTScan: A program for detecting, evaluating, and reconstructing potential coding regions in EST sequences. Proceedings of the Seventh International Conference on Intelligent Systems for Molecular Biology, 1999.

[47] Miyata T., Yasunaga T. (1980). Molecular evolution of mRNA: A method for estimating evolutionary rates of synonymous and amino acid substitutions from homologous nucleotide sequences and its application. J. Mol. Evol..

[48] Yang Z., Nielsen R. (2000). Estimating synonymous and nonsynonymous substitution rates under realistic evolutionary models. Mol. Biol. Evol..

[49] Zhang Z., Li J., Zhao X.-Q., Wang J., Wong G.K.-S., Yu J. (2006). KaKs_Calculator: Calculating Ka and Ks through model selection and model averaging. Genomics Proteomics Bioinform..

[50] Li B., Dewey C. (2011). RSEM: Accurate transcript quantification from RNA-Seq data with or without a reference genome. BMC Bioinform..

[51] Mortazavi A., Williams B.A., McCue K., Schaeffer L., Wold B. (2008). Mapping and quantifying mammalian transcriptomes by RNA-Seq. Nat. Methods.

[52] Anders S., Huber W. (2010). Differential expression analysis for sequence count data. Genome Biol..

[53] Wang L., Feng Z., Wang X., Wang X., Zhang X. (2010). DEGseq: An R package for identifying differentially expressed genes from RNA-seq data. Bioinformatics.

[54] Robinson M.D., McCarthy D.J., Smyth G.K. (2010). edgeR: A Bioconductor package for differential expression analysis of digital gene expression data. Bioinformatics.

[55] Robinson M.D., Oshlack A. (2010). A scaling normalization method for differential expression analysis of RNA-seq data. Genome Biol..

[56] Storey J.D., Tibshirani R. (2003). Statistical significance for genomewide studies. Proc. Natl. Acad. Sci. USA.

[57] Mao X., Cai T., Olyarchuk J.G., Wei L. (2005). Automated genome annotation and pathway identification using the KEGG Orthology (KO) as a controlled vocabulary. Bioinformatics.

[58] Benjamini Y., Hochberg Y. (2000). On the adaptive control of the false discovery rate in multiple testing with independent statistics. J. Educ. Behav. Stat..

[59] Francisco J.S., Moraes H.P.D., Dias E.P. (2004). Evaluation of the Image-Pro Plus 4.5 software for automatic counting of labeled nuclei by PCNA immunohistochemistry. Braz. Oral Res..

[60] Wang C.-J., Zhou Z.-G., Holmqvist A., Zhang H., Li Y., Adell G., Sun X.-F. (2009). Survivin expression quantified by Image Pro-Plus compared with visual assessment. Appl. Immunohistochem. Mol. Morphol..

